# Fatal Rickettsia Infection Presenting With Acute Respiratory Distress, Sepsis, Thrombocytopenia, and Encephalopathy: A Case Report

**DOI:** 10.7759/cureus.109360

**Published:** 2026-05-21

**Authors:** Maria A Kelley, Marianne Meshreky, Sofia Farooqui

**Affiliations:** 1 Internal Medicine, St. Mary Medical Center, Langhorne, USA

**Keywords:** doxycycline, endothelial dysfunction, fatal infections, multi-organ failure, rickettsia rickettsii, septic shock, thrombocytopenia

## Abstract

Rocky Mountain spotted fever (RMSF) is one of the most common tick-borne rickettsial diseases in the USA. It is caused by the bacterium *Rickettsia rickettsii*, which is found in most states.

It tends to be fatal even in otherwise healthy people. The diagnosis of RMSF is difficult because of the non-specific, wide-ranging presentations of the disease, especially early in its course, when symptoms may begin with a mild fever, headache, and rash after three or more days of illness, and may progress to fulminant disease. Prompt empirical antimicrobial therapy and aggressive supportive care are mandatory to avoid life-threatening complications, and treatment should not be delayed while awaiting diagnostic confirmation.

We present a case of a 62-year-old male who presented with confusion and multiple falls, later developing a maculopapular rash. The initial non-specific symptoms contributed to a delay in diagnosis. Complicating management further, the patient has a documented allergy to doxycycline, the first-line treatment for RMSF. This case underscores the importance of early clinical suspicion and the unique challenges in managing RMSF in patients with a contraindication to standard therapy.

## Introduction

Rocky Mountain spotted fever (RMSF), caused by *Rickettsia rickettsii*, is one of the most serious tick-borne illnesses in the United States. Despite being treatable, it can be rapidly fatal, especially when diagnosis is delayed. Early recognition is challenging due to its non-specific initial symptoms, which can include fever, headache, and malaise, with or without the presence of a rash. When present, the rash typically begins as small, flat, pink, non-pruritic macules that appear two to five days after the onset of fever and subsequently spread to involve the palms and soles. As the disease progresses, a petechial rash may appear, which is more commonly associated with severe illness. Progression can be swift, leading to multi-organ dysfunction and death if treatment is not promptly initiated.

Doxycycline is life-saving in RMSF and should not be delayed while awaiting diagnostic confirmation [1]. Tetracycline hypersensitivity is rarely reported, and among this class, doxycycline is the most tolerable. In our case, a reported history of doxycycline-induced anaphylaxis contributed to hesitation in initiating first-line therapy. Combined with the patient’s initial non-specific presentation, this introduced diagnostic uncertainty, and RMSF was not included among the leading differential diagnoses. As a result, broad-spectrum antibiotics were initiated empirically and unfortunately resulted in a delay in appropriate rickettsial coverage. 

## Case presentation

A 62-year-old male with a history of atrial fibrillation, hypertension, and type II diabetes mellitus presented to the Emergency Department (ED) for evaluation of confusion. His symptoms had progressively worsened over the preceding week, including generalized weakness, fevers, chills, sweats, poor appetite, and multiple falls. He developed a diffuse maculopapular rash on his torso, back, and thighs. On initial evaluation, the patient was afebrile, tachycardic, tachypneic, and borderline hypotensive. His oxygen saturation was 90%, meeting systemic inflammatory response syndrome (SIRS) criteria, as presented in Table 1. His neurological evaluation was notable for delirium and visual hallucinations but no focal neurological deficits. Mild abdominal distention with splenomegaly was noted on physical examination. Lab results are listed in Table 2.

**Table 1:** Systemic inflammatory response criteria and patient's initial findings

Criteria	Standard threshold	Patients findings	Meets criteria
Heart rate	>90 bpm	112 bpm	Yes
Respiratory rate	>20 breaths/min	32	Yes
Temperature	>38 - < 36°C	36.8°C	No
White blood cell count	>12.0 or <4.0 x 10^12^/L	4.70 x 10^12^/L	No

**Table 2:** Laboratory findings of the patient MCV: mean corpuscular volume; RDW: red cell distribution width; MPV: mean platelet volume

Lab test	Result	Normal range	Interpretation
Sodium (Na⁺)	126 mmol/L	135-145 mmol/L	Low (hyponatremia)
Potassium (K⁺)	3.6 mmol/L	3.5-5.0 mmol/L	Normal
Chloride (Cl⁻)	96 mmol/L	98-106 mmol/L	Slightly low
CO₂ (Bicarb)	21 mmol/L	22-29 mmol/L	Slightly low
Glucose	233 mg/dL	70-99 mg/dL (fasting)	High (hyperglycemia)
Creatinine	1.20 mg/dL	0.6-1.2 mg/dL	High-normal
Lactate	2.9 mmol/L	0.5-2.2 mmol/L	High (lactic acidosis possible)
Troponin	50 ng/L	<0.04 ng/mL	Markedly elevated
WBC	8.7 x 10^9^/L	4.0-11.0 x 10^9^/L	Normal
RBC	4.78 x 10^12^/L	4.5-5.9 x 10^12^/L	Normal
Hemoglobin	14.3 g/dL	13.5-17.5 g/dL (male)	Normal
Hematocrit	40.50%	41-53% (male)	Slightly low
MCV	84.7 fL	80-100 fL	Normal
RDW	13.80%	11.5-14.5%	Normal
Platelets	37 x 10^9^/L	150-450 x 10^9^/L	Severely low (thrombocytopenia)
MPV	10.7 fL	7.5-11.5 fL	Normal

The patient reported recent travel to Virginia with exposure to a wooded picnic area in late June. On admission, he denied known tick exposure. No tick, eschar, or bite lesion was identified on physical examination. 

Timeline

The patient presented on day 0 with acute-onset severe sepsis of unknown etiology and was empirically started on broad-spectrum antibiotics, including cefepime and vancomycin. Given his recent exposure to a wooded environment, there was high clinical suspicion for a tick-borne illness. Standard treatment with doxycycline was contraindicated due to a documented history of severe anaphylaxis with airway involvement following prior exposure.

An expanded infectious workup was obtained, including blood cultures, urinalysis, imaging, and serologies for tick-borne illnesses such as anaplasmosis, ehrlichiosis, and babesiosis, as well as hepatitis and HIV. A peripheral smear revealed toxic vacuolization of neutrophils without evidence of red cell inclusions, supporting a diagnosis of tick-borne illness.

Despite ongoing supportive care, the patient’s condition continued to deteriorate, with worsening agitation, hypoxia, and encephalopathy necessitating intensive care monitoring. On day 1, given the ongoing high suspicion for tick-borne disease, a rickettsial panel including polymerase chain reaction (PCR) and serologies was sent. The patient received a single dose of intravenous rifampin, and doxycycline desensitization was initiated; chloramphenicol was considered as an alternative.

On day 2, the patient rapidly deteriorated overnight with worsening encephalopathy. Due to declining mental status, the patient was endotracheally intubated for airway protection. Despite aggressive supportive management, the patient continued to decompensate and developed profound bradycardia progressing to pulselessness. A cardiac arrest code was called overhead, and advanced cardiovascular life support (ACLS) was initiated at 9:44 AM. He received atropine followed by intravenous epinephrine, along with sodium bicarbonate pushes and calcium chloride.

During resuscitation, the patient developed torsades de pointes followed by pulseless ventricular tachycardia and received 2 g of magnesium sulfate and lidocaine in addition to defibrillation at 200 J. Ventricular arrhythmia recurred, requiring two additional defibrillation attempts at 360 J. Despite prolonged resuscitative efforts and exhaustive ACLS interventions, return of spontaneous circulation was not achieved. The patient was pronounced deceased at 10:44 AM before any of the rickettsial test results were available (Table 3).

**Table 3:** Post-mortem tick-borne serology results

Test	Result
*Rickettsia* spp. DNA (real-time PCR)	Detected
Rocky Mountain spotted fever IgG	Not detected
Rocky Mountain spotted fever IgM	Not detected
Typhus fever IgG	Not detected
Typhus fever IgM	Not detected
*Ehrlichia chaffeensis* IgG	<1:64
*Ehrlichia chaffeensis* IgM	<1:20
*Babesia microti* IgG	<1:64
*Babesia microti* IgM	<1:20
*Anaplasma phagocytophilum* IgG	<1:64
*Anaplasma phagocytophilum* IgM	<1:20
Lyme antibody	<0.90

A CTA of the chest revealed interstitial pulmonary edema with small bilateral infiltrates and atelectasis (Figure 1). The imaging findings did not appear to fully correlate with the severity of the patient’s clinical presentation and rapid respiratory decline. An enlarged subcarinal lymph node was noted, likely representing reactive lymphadenopathy. No repeat imaging was obtained due to the patient’s rapid deterioration. 

**Figure 1:**
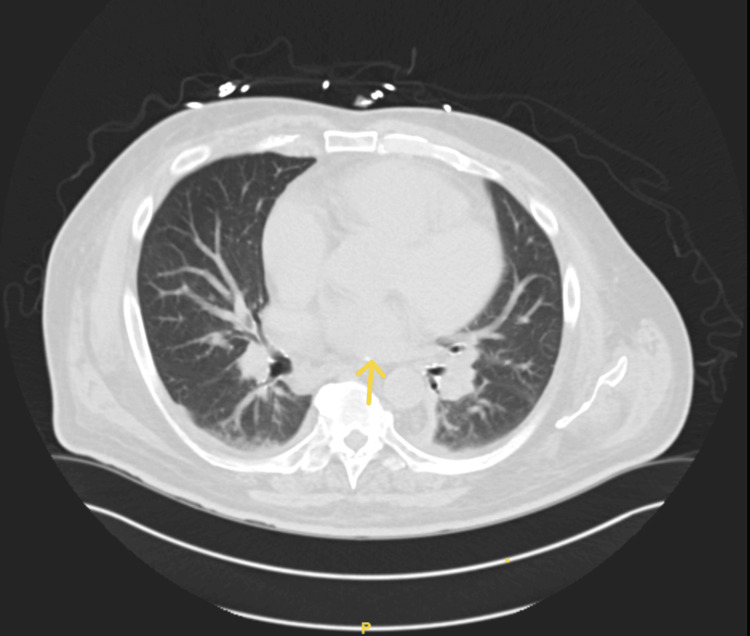
CTA chest Mild interstitial pulmonary edema with small bilateral pleural effusions and dependent atelectasis. Enlarged subcarinal lymph node (yellow arrow), likely reactive.

## Discussion

RMSF is a moderate- to severe, life-threatening multisystem disease caused by the obligate intracellular coccobacillus *R. rickettsii*. It was first described in the Rocky Mountain region in the late 1800s [1]. However, it is now reported throughout the United States; nonetheless, it is disproportionately concentrated in the South, Midwest, and South Atlantic regions, where most reported cases occur in North Carolina, Oklahoma, Arkansas, Tennessee, and Missouri, accounting for over 60% of cases [2,3]. Since the 1920s, RMSF and other rickettsial diseases have been nationally notifiable conditions. Despite advancements in early recognition, case fatality rates remain approximately 5%-10%, increasing significantly with delayed antibiotic treatment [2].

In the U.S., the primary vector of RMSF is the *Dermacentor* tick, commonly known as the American dog tick. High-risk locations include wooded, grassy, and shrubby areas, with most cases occurring in individuals exposed to tick-infested environments, particularly those with free-roaming dogs. While cases peak during summer, infections can occur from April to September. The incubation period typically ranges from 5 to 10 days, with symptoms commonly developing within the first week of infection [2].

Early recognition of RMSF is critical, as its progression can be fatal without proper treatment [1]. The clinical presentation includes fever, headache, myalgias, photophobia, abdominal pain, and nausea. *R. rickettsii* has a high affinity for endothelial cells, leading to increased vascular permeability and inflammation, manifesting as a maculopapular, petechial, or vesicular rash. While the rash is a hallmark finding, it may not always be present and typically appears three to four days after fever onset [3]. If left untreated, the disease can lead to widespread microvascular damage, capillary leakage, and platelet consumption, resulting in severe complications such as noncardiogenic pulmonary edema, cerebral edema, and disseminated intravascular coagulation (DIC) [3].

In our case, the patient developed acute respiratory distress syndrome (ARDS), thrombocytopenia, sepsis, and encephalopathy - severe manifestations that are often associated with delayed diagnosis and treatment. The rapid progression of microvascular injury and systemic inflammation led to multi-organ failure, ultimately resulting in a fatal outcome.

Diagnosis of RMSF is primarily clinical, as laboratory confirmation remains challenging. Indirect immunofluorescence assay (IFA) is considered the gold standard for diagnosis, but its sensitivity is low in the first seven days of infection, improving significantly two to three weeks after symptom onset [4-6]. Therefore, reliance on serologic testing alone should not delay appropriate treatment.

RMSF is a medical emergency in which treatment should be initiated immediately based on clinical suspicion, without awaiting confirmatory testing. Doxycycline remains the first-line treatment at a dose of 100 mg twice daily, continued for at least three days after fever resolution and clinical improvement (typically five to seven days total). Intravenous doxycycline is available for severe cases or when oral therapy is not feasible [5,7].

In patients with a reported doxycycline allergy, management is challenging because no alternative agent has equivalent efficacy. Doxycycline desensitization can be performed in urgent situations using rapidly escalating oral or intravenous doses under close monitoring, often completed within several hours to one day, allowing safe continuation of therapy. Chloramphenicol is the only historically accepted alternative, but it is rarely used in the United States due to toxicity (aplastic anemia and bone marrow suppression), inferior outcomes compared to doxycycline, and limited availability. Importantly, chloramphenicol is not routinely stocked in many hospitals, including ours, which was a key reason it was not administered in this case.

Other broad-spectrum antibiotics, including beta-lactams, aminoglycosides, and macrolides, are ineffective against *R. rickettsii* and are not recommended. While rifampin has limited in vitro activity and occasional anecdotal use, it is not considered standard therapy [6]. In our case, the patient was initially treated empirically with cefepime and rifampin due to concern for possible tick-borne illness in the setting of doxycycline allergy. However, lack of early definitive antirickettsial therapy likely contributed to rapid clinical decline, underscoring the importance of immediate initiation of doxycycline when RMSF is suspected.

## Conclusions

This case highlights the devastating consequences of RMSF when diagnosis is delayed and first-line treatment is unavailable. The combination of ARDS, thrombocytopenia, sepsis, and encephalopathy reflects the severe end of the disease spectrum. All findings emphasize the need for increased awareness of RMSF, prompt empirical doxycycline therapy, and the ongoing challenge of managing patients with contraindications to first-line treatment. Given the high mortality associated with untreated RMSF or inappropriate treatment, further research is needed to explore alternative treatment options for doxycycline-allergic patients.
